# The genus *Juglanconis* (*Diaporthales*) on *Pterocarya*

**DOI:** 10.1007/s11557-018-01464-0

**Published:** 2019-03-04

**Authors:** Hermann Voglmayr, Walter M. Jaklitsch, Hamid Mohammadi, Mohammad Kazemzadeh Chakusary

**Affiliations:** 1 Division of Systematic and Evolutionary Botany, Department of Botany and Biodiversity Research, University of Vienna, Rennweg 14, 1030 Wien, Austria; 2 Department of Forest and Soil Sciences, BOKU-University of Natural Resources and Life Sciences, Institute of Forest Entomology, Forest Pathology and Forest Protection, Franz Schwackhöfer Haus, Peter-Jordan-Straße 82/I, 1190 Vienna, Austria; 3 Department of Plant Protection, Faculty of Agriculture, Shahid Bahonar University of Kerman, Kerman, 7616914111 Iran

**Keywords:** *Ascomycota*, *Juglanconidaceae*, Molecular phylogeny, Pathogen, Systematics, 1 new name

## Abstract

Based on molecular phylogenetic analyses of a multigene matrix of partial nuSSU-ITS-LSU rDNA, *cal*, *his*, *ms204*, *rpb1*, *rpb2*, *tef1* and *tub2* sequences, recent European and Iranian collections of *Melanconium pterocaryae* from the type host, *Pterocarya fraxinifolia*, are shown to be distinct from the Japanese *Melanconis pterocaryae* from *Pterocarya rhoifolia*, and both are confirmed as closely related members of the recently described genus *Juglanconis*. Therefore, the new name *Juglanconis japonica* is proposed for *Melanconis pterocaryae*. As no type collection could be traced, *Melanconium pterocaryae* (syn. *J. pterocaryae*) is neotypified, described and illustrated, and it is recorded for Europe for the first time. During field surveys in natural stands of *P. fraxinifolia* in Guilan province (Iran), *Juglanconis pterocaryae* was consistently isolated from tissues affected by branch and trunk cankers, twig dieback and wood necrosis, indicating that it is the causal agent of these diseases. The external and internal symptoms associated with these trunk diseases are described and illustrated.

## Introduction

The Diaporthales (Ascomycota, Sordariomycetes) comprise important plant pathogens, but the species diversity and host range of many phytopathologically important lineages are still imperfectly known. Recently, substantial progress was made to tackle the species diversity of several diaporthalean lineages involved in plant diseases by the application of multi-gene phylogenies in combination with morphological studies, e.g. in *Coniella* (Alvarez et al. 2016), *Cytospora* (Lawrence et al. 2018), *Diaporthe* (Guarnaccia et al. 2018) and *Harknessia* (Marin-Felix et al. 2019). These studies revealed a number of undescribed species on various plant hosts of economic importance in silvi-, agri- and horticulture, but also improved our knowledge on the circumscription and host range of already described species.

Based on morphology and molecular phylogenies, the genus *Pterocarya* is the closest relative of the genus *Juglans* in tribe Juglandinae, Juglandaceae (Manos et al. 2007; Xiang et al. 2016). The genus *Pterocarya* currently comprises about six accepted species, of which five occur in Eastern Asia (Vietnam, China, Korea and Japan), while one species, *P. fraxinifolia*, occurs widely disjunct in Western Asia from Anatolia via the southern Caucasus area to the Caspian forest of Iran (also known as Northern Iran) and Azerbaijan (Rix 2007). In Iran, *P. fraxinifolia* grows wildly in the three northern provinces Golestan, Guilan and Mazandaran, but in recent years, small populations have also been reported in two other western provinces, Lorestan (in the Zagros Mountains) and Ilam (bordering Iraq) (Nabavi et al. 2008). For a long time, native and local people have used young leaves of this tree as an anaesthetic agent for catching fish (Sadighara et al. 2009), for dyeing and as an antifungal agent (Hadjmohammadi and Kamyar 2006; Ebrahimzadeh et al. 2008, 2009). Various parts of this plant are rich in phenolic and flavonoid compounds (Ebrahimzadeh et al. 2008; Nabavi et al. 2008) and may therefore provide interesting bioactive compounds. Although *P. fraxinifolia* is currently of little economic importance in forestry, it has been planted as an ornamental tree throughout Europe mainly in large parks (Forrest 2006). So far, although *Pterocarya* species represent important components of Western and Eastern Asian forest ecosystems and are widely planted as ornamental trees, their mycobiota are poorly known and largely understudied.

Voglmayr et al. (2017) recently described the new genus *Juglanconis* for four *Melanconis* species on hosts of tribe Juglandinae, viz. three species (*Juglanconis appendiculata*, *J. juglandina*, *J. oblonga*) on various *Juglans* species and one (*J. pterocaryae*) from *Pterocarya* spp. During these investigations, the taxonomy of *J. pterocaryae* proved to be a complex issue that could not be resolved with certainty, as it involved asexual and sexual morphs described from two different *Pterocarya* hosts, i.e. *P. fraxinifolia* and *P. rhoifolia* from Western Asia and Japan, respectively. As first species, the asexual *Melanconium pterocaryae* was described by Kuschke (1913) from *P. fraxinifolia* collected in the Georgian Republic (Abkhazia). The species apparently was not recollected again until Riedl and Ershad (1977) published a record from the same host from Iran. No sexual morph is known from this host, and no specimens or cultures were available for morphological investigations and sequencing. Based on a holomorphic collection from *P. rhoifolia* collected in Japan, Kobayashi (1970) described *Melanconis pterocaryae*, and he considered that his species represented the sexual morph of *Melanconium pterocaryae*, based on similar conidial sizes of the Japanese collection and the original description of *M. pterocaryae* by Kuschke (1913). This synonymy was also accepted by Voglmayr et al. (2017), who accordingly combined the older *Melanconium pterocaryae* into their new genus *Juglanconis*. However, at that time, this synonymy could only be based on morphological evidence, because DNA data were only available for the ex-type culture of the Japanese *Melanconis pterocaryae*, but not for isolates from *P. fraxinifolia*, the type host of the basionym.

Recently, fresh collections from the type host of *Melanconium pterocaryae*, *P. fraxinifolia*, were made in Austria, the Czech Republic and Iran. This enabled us to perform detailed morphological investigations as well as pure culture isolation for sequencing and molecular phylogenetic analyses to resolve the taxonomic status of *Melanconium pterocaryae* and *Melanconis pterocaryae*, the results of which are reported here.

## Materials and methods

### Field survey and sample collection

During 2013–2017, natural forests in Guilan province (Northern Iran) were surveyed for endophytic fungal pathogens associated with trunk diseases of *Pterocarya fraxinifolia*. Symptomatic branches (1–4 samples from each tree) from trees showing canker and dieback were collected randomly from Asalem (Talesh), Chobar (Shaft), Jirdeh (Shaft), Masal, Rezvanshar (Talesh), Rudbar, Shaft and Talesh. Cross sections of symptomatic branches were examined in order to investigate development of wood necrosis in the wood and the type of necrosis was recorded. For fungal isolations, small wood fragments (5–8 mm) were cut from the margin between healthy and affected wood tissues. Wood discs were surface disinfected by immersion in 2% sodium hypochlorite (NaOCl) for 2 min and rinsed twice in sterile distilled water (SDW). Then they were dried under sterile airflow in the laminar hood and were placed on Petri dishes containing malt extract agar (MEA: 2% malt extract, Merck, Darmstadt, Germany) supplemented with 100 mg/l streptomycin sulphate (MEAS). Petri dishes were incubated at 25 °C for 5–15 days. Growth of endophytic fungi from the tissue segments were subcultured onto fresh MEA plates and incubated at 25 °C. In most cases, cankers and twigs with dieback symptoms were covered with black conidiomata (acervuli). Fungal isolations were made also from conidiomata formed on cankers and twigs. During 2017–2018, cankered branches of *P. fraxinifolia* bearing black conidiomata were also collected in landscape parks in Austria and the Czech Republic and pure cultures isolated from conidia.

### Sample sources

Of the 12 isolates of *Juglanconis pterocaryae* from *P. fraxinifolia* included in the morphological and molecular phylogenetic analyses, 10 originated from conidia of fresh specimens and 2 were isolated from diseased host tissues (IRNHM-K116 = IRNHM-JP116 and IRNHM-K151 = IRNHM-JP151). Details of the strains including NCBI GenBank accession numbers of gene sequences used to compute the phylogenetic trees are listed in Table 1. Strain acronyms other than those of official culture collections are used here primarily as strain identifiers throughout the work. Representative isolates have been deposited at the Westerdijk Fungal Biodiversity Centre, Utrecht, The Netherlands (CBS culture collection). Details of the specimens used for morphological investigations are listed in the Taxonomy section under the respective descriptions. Herbarium acronyms are according to Thiers (2018). Specimens have been deposited in the Fungarium of the Department of Botany and Biodiversity Research, University of Vienna (WU).

**Table 1:** Strains and NCBI GenBank accessions used in the phylogenetic analyses of the combined multigene matrix of *Juglanconis*; accessions of *J. pterocaryae* for which only the ITS-LSU was sequenced were not included in the phylogenetic analyses. Sequences formatted in bold were generated during the present study

Taxon	Strain	Culture collection	Herbarium	Origin	Host	GenBank accession no.							
						ITS-LSU	*cal*	*his*	*ms204*	*rpb1*	*rpb2*	*tef1*	*tub2*
*Juglanconis appendiculata*	D140		WU 35956	Greece	*Juglans regia*	KY427138	–	–	KY427157	–	KY427188	KY427207	KY427226
	D96		WU 35954	Austria	*Juglans nigra*	KY427139	–	–	–	–	KY427189	KY427208	–
	D96A		WU 35954	Austria	*Juglans nigra*	KY427140	–	–	KY427158	–	KY427190	KY427209	–
	MC		WU 32010	Greece	*Juglans regia*	KY427141	KY427242	–	KY427159	KY427174	KY427191	KY427210	KY427227
	MC2		WU 35957	Spain	*Juglans regia*	KY427142	KY427243	–	KY427160	KY427175	KY427192	KY427211	KY427228
	MC4		WU 35958	Spain	*Juglans regia*	KY427143	KY427244	–	KY427161	KY427176	KY427193	KY427212	KY427229
	ME17, W.J.1665, A.R.3581	CBS 123194	WU 35951, BPI 840932	Austria	*Juglans regia*	KY427144	KY427245	–	KY427162	KY427177	KY427194	KY427213	KY427230
*Juglanconis juglandina*	D142		WU 35960	Austria	*Juglans regia*	KY427145	–	–	–	–	KY427195	KY427214	–
	MC1		WU 35967	Austria	*Juglans regia*	KY427146	KY427246	KY427128	KY427163	KY427178	KY427196	KY427215	KY427231
	MC3		WU 35968	Spain	*Juglans regia*	KY427147	KY427247	KY427129	KY427164	KY427179	KY427197	KY427216	KY427232
	ME16, W.J.1450, A.R.3420	CBS 121083	BPI 843622	Austria	*Juglans regia*	KY427148	KY427248	KY427130	KY427165	KY427180	KY427198	KY427217	KY427233
	ME22, W.J.1500, A.R.3860	CBS 133343	WU 35959	Austria	*Juglans regia*	KY427149	KY427249	KY427131	KY427166	KY427181	KY427199	KY427218	KY427234
	ME23		WU 35965	Austria	*Juglans nigra*	KY427150	KY427250	KY427132	KY427167	KY427182	KY427200	KY427219	KY427235
*Juglanconis oblonga*	ME14, A.R.4413	CBS 133344	–	USA	*Juglans cinerea*	KY427151	KY427251	KY427133	KY427168	KY427183	KY427201	KY427220	KY427236
	ME15, A.R.4529	CBS 133330	–	USA	*Juglans cinerea*	KY427152	KY427252	KY427134	KY427169	KY427184	KY427202	KY427221	KY427237
	ME18, M4–1	MAFF 410216	TFM FPH 2623	Japan	*Juglans ailanthifolia*	KY427153	KY427253	KY427135	KY427170	KY427185	KY427203	KY427222	KY427238
	ME19, M4–10	MAFF 410217	TFM FPH 3599, TFM FPH 3601	Japan	*Juglans ailanthifolia*	KY427154	KY427254	KY427136	KY427171	KY427186	KY427204	KY427223	KY427239
*Juglanconis japonica*	ME20, LFP-M4–8	MAFF 410079	TFM FPH 3373	Japan	*Pterocarya rhoifolia*	KY427155	KY427255	KY427137	KY427172	KY427187	KY427205	KY427224	KY427240
*Juglanconis pterocaryae*	D272	CBS 144326	WU 39981	Austria	*Pterocarya fraxinifolia*	**MK229175**	**MK238308**	**MK238312**	**MK238314**	**MK238319**	**MK238324**	**MK238332**	**MK238338**
*Juglanconis pterocaryae*	D275		WU 39983	Austria	*Pterocarya fraxinifolia*	**MK229176**	**–**	**–**	**–**	**–**	**MK238325**	**MK238333**	**–**
*Juglanconis pterocaryae*	D281		WU 39982	Austria	*Pterocarya fraxinifolia*	**MK229177**	**MK238309**	**MK238313**	**MK238315**	**MK238320**	**MK238326**	**MK238334**	**MK238339**
*Juglanconis pterocaryae*	D267a	IRNHM-JP1	WU 39985	Iran	*Pterocarya fraxinifolia*	**MK229168**	**–**	**–**	**–**	**–**	**MK238321**	**MK238329**	–
*Juglanconis pterocaryae*	D267b	IRNHM-JP8	WU 39985	Iran	*Pterocarya fraxinifolia*	**MK229169**	**–**	–	–	–	–	–	–
*Juglanconis pterocaryae*	D268a	IRNHM-JP3	WU 39986	Iran	*Pterocarya fraxinifolia*	**MK229170**	–	–	–	–	–	–	–
*Juglanconis pterocaryae*	D268b	IRNHM-JP5	WU 39986	Iran	*Pterocarya fraxinifolia*	**MK229171**	–	–	–	–	–	–	–
*Juglanconis pterocaryae*	D268c	CBS 143631 = IRNHM-JP6	WU 39986	Iran	*Pterocarya fraxinifolia*	**MK229172**	*–*	*–*	*–*	**MK238318**	**MK238322**	**MK238330**	**MK238337**
*Juglanconis pterocaryae*	D269a	IRNHM-JP4	WU 39987	Iran	*Pterocarya fraxinifolia*	**MK229173**	**–**	**–**	**–**	**–**	**MK238323**	**MK238331**	*–*
*Juglanconis pterocaryae*	D269b	IRNHM-JP7	WU 39987	Iran	*Pterocarya fraxinifolia*	**MK229174**	–	–	–	–	–	–	–
*Juglanconis pterocaryae*	K116	IRNHM-JP116	–	Iran	*Pterocarya fraxinifolia*	**MK229178**	**MK238310**	**–**	**MK238316**	**–**	**MK238327**	**MK238335**	**MK238340**
*Juglanconis pterocaryae*	K151	IRNHM-JP151	–	Iran	*Pterocarya fraxinifolia*	**MK229179**	**MK238311**	**–**	**MK238317**	**–**	**MK238328**	**MK238336**	**MK238341**
*Melanconis stilbostoma*	D143		WU 35970	Poland	*Betula pendula*	KY427156	–	–	KY427173	–	KY427206	KY427225	KY427241
*Melanconis stilbostoma*	MS	CBS 121894	**–**	Austria	*Betula pendula*	JQ926229	–	–	–	–	–	JQ926302	JQ926368

### Morphology

Microscopic observations were made in tap water except where noted. Methods of microscopy included stereomicroscopy using a Nikon SMZ 1500 equipped with a Nikon DS-U2 digital camera, and Nomarski differential interference contrast (DIC) using a Zeiss Axio Imager.A1 compound microscope equipped with a Zeiss Axiocam 506 colour digital camera. Images and data were gathered using the NIS-Elements D v. 3.22.15 or Zeiss ZEN Blue Edition software packages. Measurements are reported as maxima and minima in parentheses, and the range representing the mean plus and minus the standard deviation of a number of measurements given in parentheses. Due to poor or untypical sporulation in pure culture, conidial and conidiophore morphology was only studied in detail from natural substrates.

### Culture preparation, DNA extraction, PCR and sequencing

Single conidium isolates were prepared and grown on MEA or on 2% corn meal agar plus 2% *w*/*v* dextrose (CMD). Growth of liquid culture and extraction of genomic DNA was performed as reported previously (Voglmayr and Jaklitsch 2011; Jaklitsch et al. 2012) using the DNeasy Plant Mini Kit (QIAgen GmbH, Hilden, Germany).

The following eight loci were amplified and used for phylogenetic analyses: partial nuSSU-ITS-LSU rDNA, *cal*, *his*, *ms204*, *rpb*1, *rpb*2, *tef*1 and *tub*2; for details on loci and primers see Table 2. PCR products were purified using an enzymatic PCR cleanup (Werle et al. 1994) as described in Voglmayr and Jaklitsch (2008). DNA was cycle-sequenced using the ABI PRISM Big Dye Terminator Cycle Sequencing Ready Reaction Kit v. 3.1 (Applied Biosystems, Warrington, UK) and the PCR primers; in addition, primers ITS4, LR2R-A and LR3 were used as internal sequencing primers for the ITS-LSU rDNA region and TEF1_INTF and TEFD_iR for *tef*1 (Table 2). Sequencing was performed on an automated DNA sequencer (ABI 3730xl Genetic Analyser, Applied Biosystems).

**Table 2:** Details of the loci and primers used in the molecular study

Locus^1^	Primer	Primer sequence (5′–3′)	Orientation	Amplicon size	Reference
SSU-ITS-LSU	V9G	TTAAGTCCCTGCCCTTTGTA	Forward		de Hoog and Gerrits van den Ende (1998)
	LR5	TCCTGAGGGAAACTTCG	Reverse	ca 1.6 kb	Vilgalys and Hester (1990)
	ITS4^2^	TCCTCCGCTTATTGATATGC	Reverse		White et al. (1990)
	LR3^2^	CCGTGTTTCAAGACGGG	Reverse		Vilgalys and Hester (1990)
	LR2R-A^2^	CAGAGACCGATAGCGCAC	Forward		Voglmayr et al. (2012)
*cal*	CAL-228F	GAGTTCAAGGAGGCCTTCTCCC	Forward		Carbone and Kohn (1999)
	CAL-737R	CATCTTTCTGGCCATCATGG	Reverse	458 bp	Carbone and Kohn (1999)
*his*	CYLH3F	AGGTCCACTGGTGGCAAG	Forward		(Crous et al. (2004)
	H3-1b	GCGGGCGAGCTGGATGTCCTT	Reverse	438 bp	Glass and Donaldson (1995)
*ms204*	MSE1F1n1	AAGGGNACYCTSGAGGGCCAC	Forward		Voglmayr and Mehrabi (2018)
	MS-E5R2n	CCASAGCATGGTGGTRCCRTC	Reverse	ca 1 kb	Voglmayr and Mehrabi (2018)
*rpb*1	RPB1-Af	GARTGYCCDGGDCAYTTYGG	Forward		Stiller and Hall (1997)
	RPB1-6R1asc	ATGACCCATCATRGAYTCCTTRTG	Reverse	ca 1.2 kb	Hofstetter et al. (2007)
*rpb*2	fRPB2-5F	GAYGAYMGWGATCAYTTYGG	Forward		Liu et al. (1999)
	fRPB2-7cR	GGGGWGAYCAGAAGAAGGC	Reverse	ca 1.2 kb	Liu et al. (1999)
	dRPB2-5f	GAYACNGAYGAYCGWGAYCAYTTYGG	Forward		Voglmayr et al. (2016)
	dRPB2-7r	AANCCCATDGCYTGYTTDCCCAT	Reverse	ca 1.2 kb	Voglmayr et al. (2016)
*tef*1	EF1-728F	CATCGAGAAGTTCGAGAAGG	Forward		Carbone and Kohn (1999)
	TEF1-LLErev	AACTTGCAGGCAATGTGG	Reverse	ca 1.3 kb	Jaklitsch et al. (2005)
	TEF1_INTF^2^	CCGTGAYTTCATCAAGAACATG	Forward		Jaklitsch (2009)
	TEFD_iR^2^	GTCTGGCCATCCTTGGAGAT	Reverse		Voglmayr et al. (2018)
*tub*2	T1	AACATGCGTGAGATTGTAAGT	Forward		O’Donnell and Cigelnik (1997)
	BtHV2r	CATCATRCGRTCNGGGAACTC	Reverse	ca 1 kb	Voglmayr et al. (2017)
	T1D	CAANATGCGTGAGATTGTRAGT	Forward		This study
	T22D	CATCATRCGRTCNGGGAACTC	Reverse	ca 1.6 kb	This study

^1^*SSU-ITS-LSU*, partial nuclear 18S rDNA, internal transcribed spacers and intervening 5.8S rDNA and 28S rDNA amplified and sequenced as a single fragment; *cal*, calmodulin; *his*, histone H3; *ms204*, guanine nucleotide-binding protein subunit beta; *rpb1*, DNA-directed RNA polymerase II largest subunit; *rpb2*, DNA-directed RNA polymerase II second largest subunit; *tef*1, translation elongation factor 1-alpha; *tub*2, β-tubulin

^2^Internal sequencing primers

### Data analysis

The newly generated sequences were aligned to the sequence alignments of Voglmayr et al. (2017), and a combined matrix of the eight loci (partial SSU-ITS-LSU rDNA, *cal*, *his*, *ms204*, *rpb*1, *rpb*2, *tef1* and *tub*2) was produced for phylogenetic analyses, with two accessions of *Melanconis stilbostoma* added as the outgroup. The GenBank accession numbers of sequences used in these analyses are given in Table 1.

Sequence alignments for phylogenetic analyses were produced with the server version of MAFFT (http://mafft.cbrc.jp/alignment/server/), checked and refined using BioEdit v. 7.2.6 (Hall 1999). The combined data matrix contained 8441 characters; viz. 1600 nucleotides of SSU-ITS-LSU, 460 nucleotides of *cal*, 449 nucleotides of *his*, 1037 nucleotides of *ms204*, 711 nucleotides of *rpb*1, 1160 nucleotides of *rpb*2, 1400 nucleotides of *tef*1 and 1624 nucleotides of *tub*2.

Maximum parsimony (MP) analyses were performed with PAUP v. 4.0a163 (Swofford 2002). All molecular characters were unordered and given equal weight; analyses were performed with gaps treated as missing data; the COLLAPSE command was set to MINBRLEN. MP analysis of the combined multilocus matrix was done using 1000 replicates of heuristic search with random addition of sequences and subsequent TBR branch swapping (MULTREES option in effect, steepest descent option not in effect). Bootstrap analyses with 1000 replicates were performed in the same way, but using 10 rounds of random sequence addition and subsequent branch swapping during each bootstrap replicate.

Maximum likelihood (ML) analyses were performed with RAxML (Stamatakis 2006) as implemented in raxmlGUI 1.5 (Silvestro and Michalak 2012), using the ML + rapid bootstrap setting and the GTRGAMMA substitution model with 1000 bootstrap replicates. The matrix was partitioned for the different gene regions.

## Results

### Field survey and isolation

**Fig. 1:**
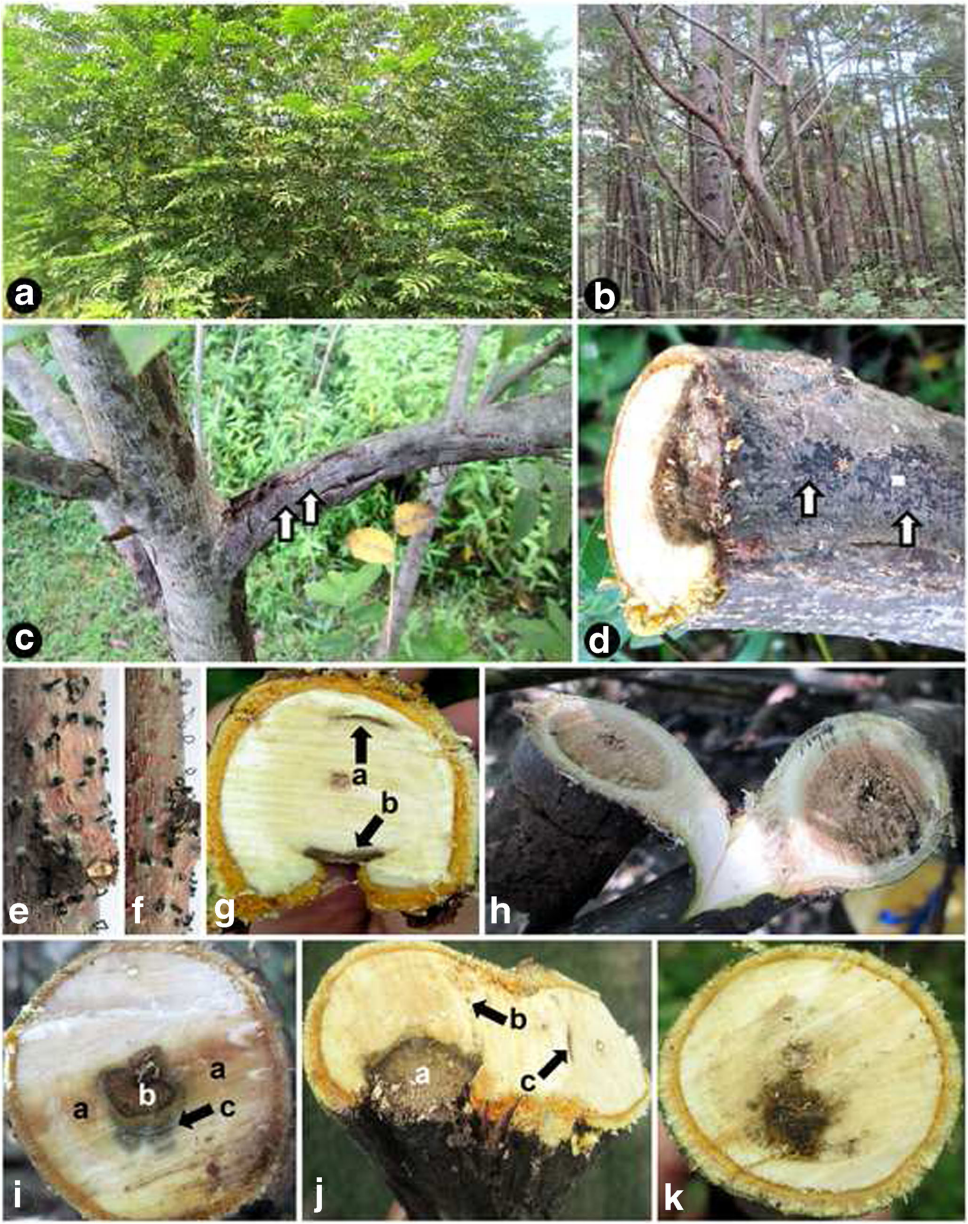
External and internal symptoms associated with trunk diseases of *Pterocarya fraxinifolia* in Asalem and Talesh (Guilan province, Northern Iran), from which *Juglanconis pterocaryae* was isolated. **a** Healthy tree. **b** Trees showing severe decline symptoms. **c** Tree showing canker and branch dieback covered by acervuli of *J. pterocaryae* (arrows). **d** Cross section of a branch showing wedge-shaped necrosis, arrows showing acervuli of *J. pterocaryae*. **e**, **f** Dead branches with *J. pterocaryae* acervuli, some with conidial cirrhi (spore tendrils). **g** Co-occurrence of arch-shaped necrosis (a) and young wedge-shaped necrosis (b). **h** Extensive central necrosis. **i** Co-occurrence of watery necrosis (a), irregular necrosis (b) and black wood streaking (c). **j** Co-occurrence of wedge-shaped necrosis (a), black spots (b) and arch-shaped necrosis (c). **k** Irregular wood necrosis

In the field surveys in the natural forests in Guilan province (Iran), declining trees of *P. fraxinifolia* showed branch and trunk canker, extensive dieback of terminal and lateral branches and death (Fig. 1b, c). Examination of branches from symptomatic trees revealed seven types of wood discolouration in cross sections: brown to black wood streaking, black spots, arch-shaped necrosis, central necrosis, irregular wood necrosis, water necrosis and wedge-shaped necrosis (Fig. 1g–k). Some collected samples showed multiple lesion types on the same sample in cross sections (Fig. 1g, i, j). A fungus morphologically resembling the genus *Juglanconis* (Voglmayr et al. 2017) was consistently isolated from wood lesions of affected trees (eight isolates). Among those isolates, seven (i.e. one from each different wood lesion type) were selected as representative isolates for further detailed studies. All of these isolates showed the same pure culture, conidioma and conidial characters. Two of these isolates, IRNHM-JP116 and IRNHM-JP151, were also selected for molecular studies. IRNHM-JP116 was isolated from infected tissue of a tree from Masal showing dieback and irregular wood necrosis in cross section, while IRNHM-JP151 was isolated from a tree from Asalem (Talesh) showing branch canker and irregular wood necrosis in cross section. During this work, 24 Iranian and three Austrian isolates were also recovered from conidiomata produced on twigs showing dieback (Fig. 1d–f). All these isolates had the same pure culture, conidioma and conidial characters like the isolates from lesions. In addition to *Juglanconis*, two isolates of *Phaeoacremonium alvesii* (Kazemzadeh Chakusary et al. 2017) and five isolates of *Lasiodiplodia mahajangana* (Kazemzadeh Chakusary et al. 2019) were isolated from affected trees. The field observations indicate that *J. pterocaryae* plays a major role in the decline of *P. fraxinifolia* in the forests of Northern Iran.

### Molecular phylogeny

**Fig. 2:**
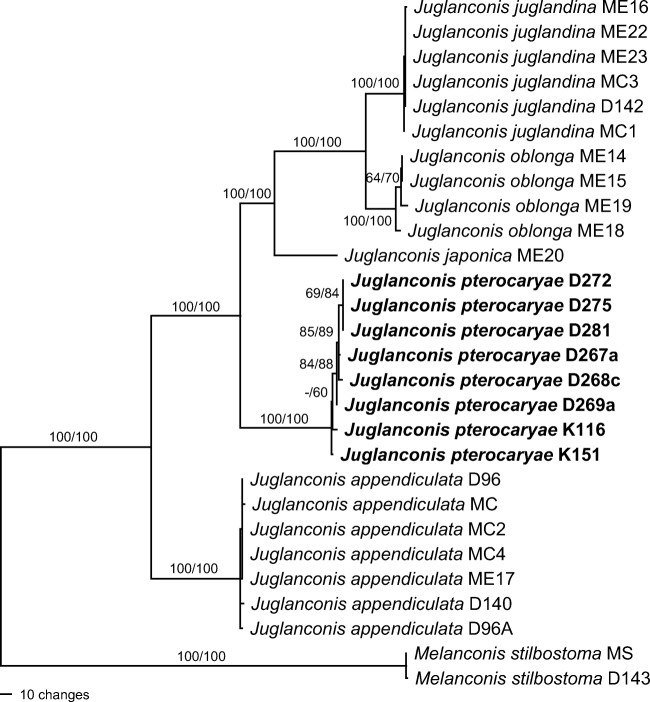
Phylogram showing one of 30 MP trees of 1090 steps (CI = 0.945, RI = 0.978, RC = 0.924) revealed by PAUP from an analysis of the combined SSU-ITS-LSU-*cal*-*his*-*ms204*-*rpb1*-*rpb2*-*tef1*-*tub2* matrix of *Juglanconis*, with *Melanconis stilbostoma* selected as outgroup. MP and ML bootstrap support above 50% are given at the first and second position, respectively, above or below the branches. Strain numbers are given following the taxon names; strains formatted in bold were isolated and sequenced in the present study

The combined multilocus matrix used for phylogenetic analyses comprised 8441 characters, of which 748 were parsimony informative (112 from SSU-ITS-LSU, 41 from *cal*, 34 from *his*, 64 from *ms204*, 35 from *rpb*1, 178 from *rpb*2, 173 from *tef*1 and 111 from *tub*2). The MP analysis revealed 30 MP trees 1090 steps long, one of which is shown in Fig. 2. Tree topologies of all MP trees were identical except for minor differences within *Juglanconis appendiculata* and *J. pterocaryae*. The ML tree revealed by RAxML was identical to the MP tree shown. *Melanconis pterocaryae* from *P. rhoifolia* and *J. pterocaryae* from *P. fraxinifolia* were revealed as distinct species; the two species were not closest relatives, but the latter was placed basal to the clade containing *M. pterocaryae*, *J. juglandina* and *J. oblonga* with maximum support. Due to the same species epithet, a new name needs to be proposed for *Melanconis pterocaryae*. All five species of *Juglanconis* received maximum support in both analyses, as well as the relationships between the species.

## Taxonomy

*Juglanconis japonica* (Tak. Kobay.) Voglmayr & Jaklitsch, nom. nov.

MycoBank: MB 828925.

Replaced synonym. *Melanconis pterocaryae* Tak. Kobay., Bull. Govt Forest Exp. Stn Meguro 226: 24. 1970, non *Melanconium pterocaryae* Kuschke, Trudy Tiflissk. Bot. Sada 28: 25. 1913.

Etymology: referring to its occurrence in Japan.

Holotype: Japan, Shizuoka, Fuji, on corticated twigs of *Pterocarya rhoifolia*, 5 Aug. 1968, T. Kobayashi (TFM FPH2623!); ex-type culture MAFF 410079.

Notes: When describing *Melanconis pterocaryae* from *P. rhoifolia* collected in Japan, Kobayashi (1970) considered his species to represent the sexual morph of *Melanconium pterocaryae* from *P. fraxinifolia*, based on similar conidial sizes. This synonymy was also accepted by Voglmayr et al. (2017), who combined the older *Melanconium pterocaryae* into the new genus *Juglanconis*. However, the current molecular phylogenies reveal *Melanconis pterocaryae* to represent a clearly distinct species, which therefore needs a new name. Morphologically, the conidial size of *J. japonica* is similar to that of *J. pterocaryae*, with slightly narrower conidia (11–20 × 5–9 μm vs. 11–22 × 6–11 μm in *J. pterocaryae*); however, the conidia of *J. japonica* usually have in average a distinctly higher length/width ratio, (1.5–)2.0–2.5(−3.1), vs. (1.3–)1.5–2.1(−3.0) in *J. pterocaryae*. For a detailed description and illustrations of the holomorph of *J. japonica* from the holotype, see Voglmayr et al. (2017; as *J. pterocaryae*).

**Fig. 3:**
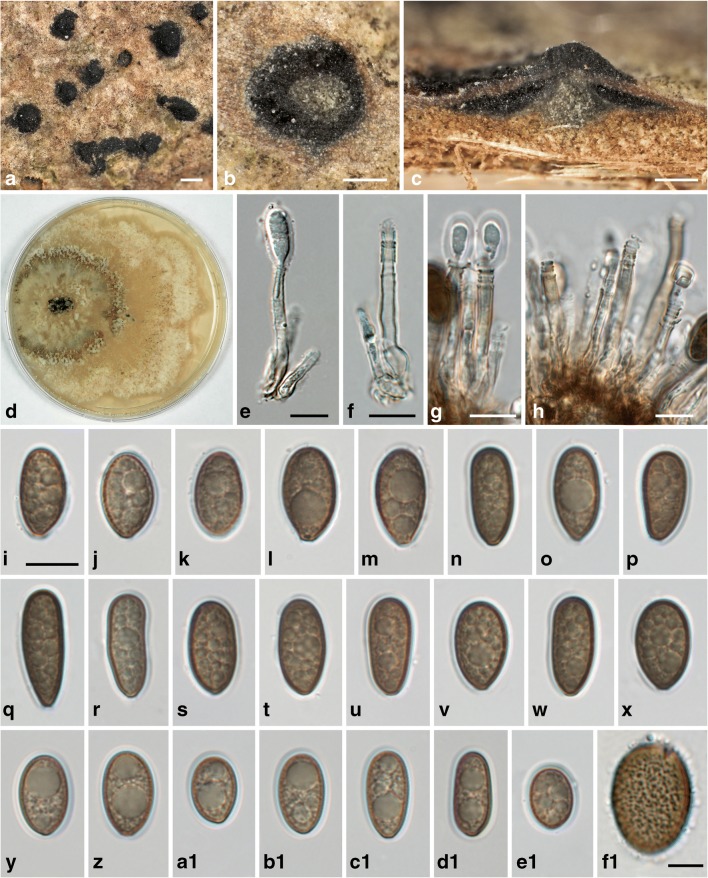
*Juglanconis pterocaryae*. **a** Conidiomata in surface view. **b**, **c** Transverse (**b**) and vertical (**c**) sections of conidiomata, showing central column. **d** Culture (CMD, 25 d, 16 °C). **e**–**h** Conidiophores (annellides; in **e**, **g** with young conidia). **i**–**e**1 Vital conidia with gelatinous sheath. **f**1 Squashed conidium showing the densely verruculose inner conidial wall. All in water (**a**–**c**, **i**–**m**, **f**1 WU 39981, neotype; **d** WU 39983; **e**, **f**, **n**–**x** WU 39982; **g**, **h** WU 39985b; **y**–**d**1 WU 39986b; **e**1 WU 39987a). Scale bars **a** 500 μm; **b**, **c** 200 μm; **e**–**e**1 10 μm; **f**1 5 μm

*Juglanconis pterocaryae* (Kuschke) Voglmayr & Jaklitsch, in Voglmayr, Castlebury & Jaklitsch, Persoonia 38: 150 (2017), emend. Fig. 3.

Basionym. *Melanconium pterocaryae* Kuschke, Trudy Tiflissk. Bot. Sada 28: 25. 1913.

*Sexual morph* unknown. *Conidiomata* on natural substrate acervular, 0.8–2.2 mm diam, embedded in bark tissues, blackish, inconspicuous, scattered, with central or eccentric conical olivaceous grey stromatic column 300–850 μm wide at the base; at maturity covered by blackish discharged conidial masses forming black spots 0.2–2.5 mm diam or sometimes long cirrhi on the cortex. *Conidiophores* (11–)17–30(−48) × (3.0–)3.5–4.7(−5.5) μm (*n* = 74), narrowly cylindrical, simple or branched at the base, smooth, subhyaline to pale brown. *Conidiogenous cells* annellidic with distinct annellations, integrated. *Conidia* (11.2–)13.3–16.8(−22.3) × (6.0–)7.5–9.3(−11.0) μm, l/w = (1.3–)1.5–2.1(−3.0) (*n* = 980), unicellular, hyaline when immature, medium to dark brown when mature, variable in shape, ellipsoid to elongate, sometimes pip-shaped, often truncate with an abscission scar at the base, densely multiguttulate, thick-walled; wall ca. 0.5–0.8 μm, with distinct ornamentation on the inside of the wall consisting of small irregular confluent verrucae 0.3–0.7 μm diam, with ca. 0.5–1 μm wide gelatinous sheath.

Culture: Colony on CMD at 22 °C reaching 70 mm diam after 7 days; first white, turning cream to greyish brown in the centre, with irregular concentric zones and tufts of woolly aerial mycelium, margin uneven, wavy. Conidial pustules formed on tufts of aerial mycelium after ca 3 weeks, up to 4 mm diam, containing numerous branched conidiophores produced on subhyaline to brown aerial hyphae. Conidia similar to those produced on natural substrate except for slightly smaller size, (8.2–)10.5–13.0(−15.2) × (5.5–)6.8–8.2(−8.8) μm, l/w = (1.2–)1.4–1.8(−2.2) (*n* = 67).

Habitat and host range: Dead corticated trunks, twigs and branches of *Pterocarya fraxinifolia*.

Distribution: Europe and Western Asia (known from Austria, Czech Republic, Georgian Republic, Iran).

Typification: Austria, Oberösterreich, Bad Hall, Kurpark, on corticated twigs of *Pterocarya fraxinifolia*, 20 Oct. 2017, W. Jaklitsch (WU 39981, neotype of *Melanconium pterocaryae* here proposed; ex neotype culture D272 = CBS 144326).

Additional specimens examined (all on corticated twigs of *Pterocarya fraxinifolia*): Austria, Niederösterreich, Bruck an der Leitha, Harrachpark, 25 Mar. 2018, H. Voglmayr (WU 39982; culture D281); Steiermark, Graz, Geidorf, Botanical Garden of the University of Graz (HBG), 5 Feb. 2018, H. Voglmayr (WU 39983; culture D275). Czech Republic, Morava, Lednice landscape park, 1 May 2018, H. Voglmayr (WU 39984). Iran, Shaft, Chobar, 28 Apr. 2017, H. Mohammadi (WU 39988); Shaft, Jirdeh, 25 Apr. 2017, H. Mohammadi (WU 39985a, b; cultures D267a, b); Talesh, Rezvanshar, 2 May 2017, M. Kazemzadeh Chakusary (WU 39986a, b, c; cultures D268a, b, c = CBS 143631); Talesh, 2 May 2017, M. Kazemzadeh Chakusary (WU 39987a, b; cultures D269a, b).

Notes: The basionym, *Melanconium pterocaryae*, was described by Kuschke (1913) from the Georgian Republic (Abkhazia) from *P. fraxinifolia*, but until recently, no collections from the original host were available for morphological investigations and for DNA sequencing, and therefore no material from that host could be included in the investigations of Voglmayr et al. (2017). The conidial sizes given in the protologue of *Melanconium pterocaryae* (14–19 × 8–12 μm) are slightly wider than those revealed in the current study (11–22 × 6–11 μm), which is in line with Riedl and Ershad (1977), who also reported narrower conidia (12–15.5 × 6.5–9.5 μm) in their Iranian collection. The conidial size and shape of *J. pterocaryae* can be quite variable between collections but also within the same specimen, probably depending on the environmental conditions during development; we observed slightly smaller conidia in the Iranian collections ((11.2–)12.0–15.5(−19.2) × (6.0–)7.5–9.0(−10.8) μm, l/w = (1.3–)1.5–1.9(−2.6) (*n* = 567)) than in the Central European ones ((11.5–)14.5–17.8(−22.3) × (6.3–)7.8–9.5(−11.0) μm, l/w = (1.3–)1.6–2.2(−3.0) (*n* = 413)). However, as the sequences of the Central European and Iranian collections are (almost) identical, this variation is confirmed to represent intraspecific variability. In contrast to the other described *Juglanconis* species, no sexual morph is known for *J. pterocaryae*.

Despite extensive enquiries, no type collection of *Melanconium pterocaryae* could be traced in Russian or Georgian herbaria. In the apparent lack of an extant type, we here propose a well-developed Austrian collection, for which a culture and sequences are available, as neotype. Although the neotype collection does not originate from the area from where the species was described, we consider this justified, as the *P. fraxinifolia* accessions (and therefore also its associated *Juglanconis*) grown in Central Europe likely originate from the Caucasus area, the conidial sizes of the neotype collection and the protologue agree well, and the conspecific Austrian and Iranian *Juglanconis* accessions confirm a wide distribution of the species that likely corresponds with the distribution of its host.

## Discussion

Previous molecular phylogenetic analyses had shown that *Melanconis* species on *Juglans* and *Pterocarya* form a highly supported lineage that is distinct from *Melanconis* sensu stricto, and the new genus *Juglanconis* was established for them (Voglmayr et al. 2017), which was classified in the new family Juglanconidaceae. However, in this previous study, only a single Eastern Asian isolate from *Pterocarya rhoifolia* could be included, but none from the Western Asian *P. fraxinifolia*. The current molecular phylogenetic analyses (Fig. 2) clearly show that *Juglanconis* accessions from *P. fraxinifolia* and *P. rhoifolia* represent two distinct species, *J. pterocaryae* and *J. japonica*, respectively. This is not surprising, as high host specificity in combination with vicariant speciation has been commonly reported in Diaporthales on woody hosts, e.g. in *Coryneum* (Jiang et al. 2018), *Cryptosporella* (Mejía et al. 2008, 2011a), *Melanconiella* (Voglmayr et al. 2012), *Melanconis* (Fan et al. 2016), *Plagiostoma* (Mejía et al. 2011b; Walker et al. 2014), *Stegonsporium* and *Stilbospora* (Voglmayr and Jaklitsch 2008, 2014). In many of these lineages, morphological species identification can be difficult due to lack of a clear morphological distinction, while molecular data but also host ranges are highly diagnostic on the species level. However, in the *Juglanconis* species on *Juglans*, host specificity was shown to be rather on the genus than on the species level, as both European species, *J. appendiculata* and *J. juglandina*, were reported from various hosts (the indigenous *Juglans regia* as well as the naturalised North American *J. nigra*), and the North American and Eastern Asian *Juglanconis oblonga* was likewise confirmed to occur on several *Juglans* species. It remains unclear whether the *Juglanconis* species on *Pterocarya* potentially have wider host ranges, their different host ranges and geographic areas being rather the result of the highly disjunct distribution of their hosts than of host specificity. Interestingly, *Melanconis*/*Melanconium* spp. have also been recorded from China on *Pterocarya stenoptera* (Farr and Rossman 2018), which has a wide distribution in Eastern Asia, occurring in China, Korea and Japan and is also widely cultivated as a shade tree (Lu et al. 1999). Investigation of isolates from this host could help to shed light on this question.

According to Kazemzadeh Chakusary (2017), *J. pterocaryae* is suspected to be one of the most important fungal agents of *P. fraxinifolia* dieback in Guilan province in Northern Iran. Seven kinds of wood lesions were associated with *P. fraxinifolia* showing decline symptoms in Iran. Similar observations were reported in previous studies conducted on trunk diseases of fruit (Van Niekerk et al. 2011, Cloete et al. 2011, Sami et al. 2014) and ornamental and forest trees (Hashemi and Mohammadi 2016; Kazemzadeh Chakusary et al. 2017). Iranian isolates were recovered from all kinds of wood lesions recorded on *P. fraxinifolia*. Moreover, a large number of acervuli of *J. pterocaryae* were observed on the surface of cankers and twigs showing dieback symptoms. During this study, several Iranian isolates of *J. pterocaryae* were isolated from necrotic wood tissues of *P. fraxinifolia* trees. We did not determine the pathogenicity of these isolates on this woody plant. Therefore, pathogenicity studies will be necessary to evaluate and confirm the importance of this species in trunk diseases of *P. fraxinifolia*.

It is remarkable that *J. pterocaryae* has apparently not been previously reported from Europe, considering its conspicuous symptoms which are similar to those of the well-known black pustular dieback disease of walnut (*Juglans*) species caused by closely related *Juglanconis* species (Graves 1923; Belisario 1999). This may be due to the fact that, compared to *Juglans* spp., *Pterocarya fraxinifolia* has little economic impact and is rather infrequently grown, mainly in botanical gardens, arboreta and large landscape parks. In one Austrian site (Harrachpark), it was found abundantly on large cut as well as recently wind-broken branches, the ejected conidial pustules covering their entire length. This indicates that *J. pterocaryae*, like other *Diaporthales*, may be commonly present as a latent pathogen in living host tissues, enabling a massive development following the death of the host tissue. *Juglanconis pterocaryae* represents another example of a tree pathogen co-occurring with its hosts in old arboreta and parks far outside their natural distribution; similar cases were, e.g. reported for North American and Southern European *Stegonsporium* spp. following their maple (*Acer*) hosts grown in Central and Western European parks (Voglmayr and Jaklitsch 2014). As these pathogens can have a long latent phase in living host tissue, they are difficult to detect and can be distributed over wide distances with the transport of symptomless but yet infected living trees. Therefore, parks and arboreta are a potential source for the introduction and establishment of alien fungal diseases of trees, and should therefore be regularly monitored especially for problem pathogens of forest trees.
